# Efficacy and safety of intravitreal injection of conbercept for moderate to severe nonproliferative diabetic retinopathy

**DOI:** 10.3389/fmed.2024.1394358

**Published:** 2024-05-23

**Authors:** Lu Shen, Yuxiang Zheng, Zelan Gao, Qirui Li, Min Dai, Wenchang Yang, Qiying Zhang, Dongli Li, Yijun Hu, Ling Yuan

**Affiliations:** ^1^Department of Ophthalmology, The First Affiliated Hospital of Kunming Medical University, Kunming, China; ^2^Department of Ophthalmology, Yan’an Hospital of Kunming City, Kunming, China; ^3^Guangdong Eye Institute, Department of Ophthalmology, Guangdong Provincial People’s Hospital (Guangdong Academy of Medical Sciences), Southern Medical University, Guangzhou, China

**Keywords:** moderate to severe nonproliferative diabetic retinopathy, Diabetic Retinopathy Severity Scale, conbercept, VEGF – vascular endothelial growth factor, diabetic macular edema

## Abstract

**Purpose:**

This study aimed to assess the effectiveness and safety of intravitreal injection of conbercept (IVC) in treating moderate to severe nonproliferative diabetic retinopathy (NPDR), with or without accompanying diabetic macular edema.

**Methods:**

In this longitudinal retrospective study, 35 patients (50 eyes) with moderate to severe NPDR and Diabetic Retinopathy Severity Scale (DRSS) scores between 43 and 53 were treated at the Department of Ophthalmology, First Affiliated Hospital of Kunming Medical University, from October 2018 to January 2023. Treatment protocol included three monthly IVC injections followed by a *pro re nata* (PRN) regimen over a two-year follow-up period. Outcome measures were best-corrected visual acuity (BCVA), intraocular pressure, central macular thickness (CMT), extent of hard exudate (HE), and changes in DRSS scores. DRSS scores before and after treatment were analyzed using the Wilcoxon rank-sum test. Both systemic and ocular adverse events were meticulously documented to ascertain safety.

**Results:**

From baseline to the final follow-up, the mean BCVA improved from 0.41 ± 0.39 to 0.23 ± 0.20 logMAR (*p*<0.05). The mean CMT decreased from 306.22 ± 77.40 to 297.97 ± 88.15 μm (*p* = 0.385). At 24 months, DRSS scores improved by ≥1 stage in 40 eyes (80%), ≥ 2 stages in 28 eyes (56%), ≥3 stages in 10 eyes (20%), and remained stable in 6 eyes (12%). The DRSS scores at each follow-up interval demonstrated statistically significant improvement from baseline (*p*<0.05). In 15 of 27 eyes (55.56%) with diabetic macular edema (DME), there was a significant reduction in the mean area of HE from baseline (*p*<0.05). No serious systemic adverse events were observed.

**Conclusion:**

IVC is an effective and safe treatment for moderate to severe NPDR, demonstrating significant improvements in DRSS scores.

## Introduction

Diabetes mellitus (DM) is projected to affect 592 million people globally by 2035, with approximately 34.6% of these individuals developing ocular microvascular complications, notably diabetic retinopathy (DR). DR is a primary cause of vision loss among working-age adults (1) and is classified into two stages: NPDR and proliferative diabetic retinopathy (PDR), based on the presence of retinal neovascularization. NPDR, the initial stage, is marked by features such as venous beading, microaneurysms, hard exudates, and cotton wool spots, and may progress to PDR, which poses a risk of severe and irreversible vision loss or total blindness due to complications like vitreous hemorrhage, tractional retinal detachment, and neovascular glaucoma.

Panretinal laser photocoagulation (PRP) is the predominant treatment for severe, late-stage NPDR. While effective at preserving visual acuity, PRP offers limited potential for long-term vision improvement and is associated with substantial adverse effects including permanent peripheral visual field loss, night blindness (nyctalopia), and exacerbation of macular edema. Furthermore, PRP may cause thinning of the retinal nerve fiber layer (2), and long-term complications such as laser scar expansion, secondary choroidal neovascularization, subretinal fibrosis, and decreased subfoveal choroidal thickness and blood flow (3). These risks underscore the need for strategies that more effectively safeguard visual function in the treatment of NPDR. To date, no studies have demonstrated that PRP can enhance the Diabetic Retinopathy Severity Scale (DRSS) scores in patients with NPDR who do not have DME.

Intravitreal injection of anti-vascular endothelial growth factor (VEGF) drugs currently serves as the first-line treatment for DME (4). Conbercept (Lumitin, Chengdu Kanghong Biotech Co., Ltd., P. R. China) is a 141-kDa recombinant anti-VEGF fusion protein, produced from full human cDNA sequence in Chinese hamster ovary cells. It has been widely utilized in clinical settings (5–7). Research has substantiated the effectiveness of conbercept in managing DME (8, 9) and PDR (10, 11). Initial reports of DRSS score improvements post-anti-VEGF injections emerged from clinical trials focusing on DME. A *post hoc* analysis of the RIDE (ClinicalTrials.gov identifier NCT00473382) and RISE (ClinicalTrials.gov identifier NCT00473330) trials (12) revealed that patients with moderate to severe NPDR (baseline DRSS score 47–53) experienced a 78% rate of improvement by two or more steps. Notably, the most marked improvements in DR severity were observed in patients with moderate to severe NPDR at baseline; these improvements was rapid, clinically significant, and sustained over 36 months. Additionally, growing evidence suggests that anti-VEGF therapy can elevate DRSS scores in patients with NPDR but without DME. The PANORAMA study, a significant prospective trial of the anti-VEGF era, targeted eyes with NPDR (DRSS 47–53) lacking DME (13). Protocol W, an ongoing phase III trial, investigates the use of aflibercept versus sham in preventing PDR or center-involving DME in eyes with NPDR (DRSS 47–53) without DME (14). These findings have prompted recommendations for anti-VEGF therapy in early-stage DR, given its potential to reverse DR grading and enhance visual acuity, all while avoiding significant retinal damage, thus potentially offering a preferable alternative to PRP for NPDR treatment. However, the specific impact of conbercept on NPDR regression remains underexplored. In this retrospective clinical study, we assessed the safety, effectiveness, and extent of DR regression, as measured by DRSS scores, following intravitreal conbercept injections in patients with early-stage moderate to severe NPDR. The findings are crucially important in preventing long-term vision loss, averting severe blindness-related complications, and diminishing the social and economic impacts of DR treatment.

## Patients and methods

### Patients

Patients were enrolled from the clinical centers at the First Affiliated Hospital of Kunming Medical University, Yunnan Province, China, between October 2018 and January 2023. The study was conducted in accordance with the Declaration of Helsinki and received approval from the Ethics Review Committee of the First Affiliated Hospital of Kunming Medical University. Due to its retrospective nature, the requirement for informed consent was waived by the Committee.

The study enrolled adults (age ≥ 18 years) diagnosed with type 1 or 2 diabetes, moderate to severe NPDR (Early Treatment Diabetic Retinopathy Study severity level, 43–53) (15), with or without DME, as assessed by the investigator. These patients maintained stable glucose levels, indicated by a glycosylated hemoglobin level of <10% throughout the follow-up period. They underwent comprehensive monthly ophthalmic examinations over 2 years, which included assessments of best-correct visual acuity (BCVA), slit-lamp biomicroscopy, intraocular pressure (IOP), and coherence tomography (OCT) using the 6 × 6 mm Retina Angio procedure (RTVue XRAvanti, Optovue, Inc., Fremont, CA, United States). Ultrawide-field fundus photography was performed using the Optomap Panoramic Daytona device (Daytona, Optos, United Kingdom). DRSS levels were categorized as follows: DR absent, level 10; minimal DR (microaneurysms only), level 20; mild NPDR, level 35; moderate NPDR, level 43; moderately severe NPDR, level 47; severe NPDR, level 53; mild PDR, level 61; moderate PDR, level 65; and high-risk PDR, level 71/75. DRSS scores were consistently evaluated by the same specialist (L.Y.). Binary segmentation images were produced using automatic threshold functions and manual adjustments on the Image J software (Rasband, W.S., ImageJ, U. S. National Institutes of Health, Bethesda, Maryland, United States). The area of hard exudation (HE) lesion within the macular vascular arch was measured semi-automatically in fundus images taken before and during the follow-up periods. Data were systematically recorded prior to treatment and at intervals of 1, 2, 3, 6, 9, 12, 18 and 24 months after the initial treatment.

Patients were excluded if they had undergone vitreoretinal surgery or panretinal photocoagulation, or if they had histories of vitreoretinal traction, vitreous hemorrhage, uveitis, uncontrolled glaucoma, macular fibrovascular proliferation, or significant media opacities. Additional exclusion criteria included other retinal pathologies such as high myopia, age-related macular degeneration, or systemic conditions like untreated or uncontrolled hypertension. Safety was monitored by documenting any severe adverse events such as endophthalmitis, vitreous hemorrhage, or cerebrovascular disease.

### Treatment

All participants received intravitreal injections of conbercept (0.5 mg/0.05 mL) following a 3 + *pro re nata* (PRN) regimen (16–18), administered under aseptic conditions. This regimen consisted of one injection per month for the first 3 months, followed by additional treatments based on clinical need. PRN criteria were defined as follows: (a) For patients with DME, treatment was initiated if the CMT increased by 10% or more from baseline accompanied by a 5- to 9-letter decrease in visual acuity at two consecutive visits, attributable to DME; (b) For patients without DME, treatment was considered if there was an exacerbation of one or more DRSS levels. Rescue therapy included focal retinal laser photocoagulation if areas of retinal non-perfusion were observed. Furthermore, 25G vitrectomy combined with PRP and IVC was performed if PDR developed, indicated by complications such as vitreous hemorrhage or traction retinal detachment. Primary outcome measures included changes in DRSS levels, with secondary outcomes being changes in BCVA, CMT, and HE.

### Statistical analysis

Data analyses were conducted using SPSS Statistics for Windows, version 27.0 (IBM Corp., Armonk, NY, United States) and GraphPad Prism software, version 9.0 (GraphPad Software, Inc., SanDiego, California, United States). Measurement data are presented as mean ± standard deviation. The paired-sample *t*-test was used to assess changes in BCVA and CMT throughout the follow-up period. The Wilcoxon signed-rank test was applied for ranked data comparisons. A *p* value of ≤0.05 was deemed statistically significant.

## Results

### Patient clinical characteristics and baseline statistics

The study evaluated 50 eyes from 35 patients with NPDR (16 males and 19 females; 24 right eyes and 26 left eyes). Baseline characteristics are described in Table 1. The mean age was 56.56 ± 11.72 years, and the average duration of DM was 18.33 ± 6.62 years. DME was present in 27 eyes, while 23 eyes did not have DME, distributed as follows: 15 moderate NPDR, 18 moderately severe NPDR, and 17 severe NPDR. The initial mean BCVA was 0.41 ± 0.39 logMAR, and the initial mean CMT was 306.22 ± 77.40 μm. Patients received an average of 8.15 ± 2.97 injections during the follow-up period.

**Table 1:** Baseline patient demographics and ocular characteristics.

Parameters	
Patients/eyes (*n*)	35/50
Sex (*n*, male/female)	16/19
Eyes (*n*, right/left)	24/26
Mean age (years, mean ± standard deviation)	56.56 ± 11.72
Mean duration of diabetes (years, mean ± standard deviation)	18.33 ± 6.62
DME (*n*, with/without)	27/23
Chronic kidney insufficiency (*n*)	6
Stage of DR (*n*)	
Moderate NPDR	15
Moderately severe NPDR	18
Severe NPDR	17
Baseline BCVA (logMAR, mean ± standard deviation)	0.41 ± 0.39
Baseline CMT (μm, mean ± standard deviation)	306.22 ± 77.40
Number of injections (mean ± standard deviation)	8.15 ± 2.97

DME, diabetic macular edema; DR, diabetic retinopathy; NPDR, nonproliferative diabetic retinopathy; BCVA, best-corrected visual acuity; CMT, central macular thickness; logMAR, logarithm of the minimum angle of resolution.

### Changes in BCVA

**Figure 1:**
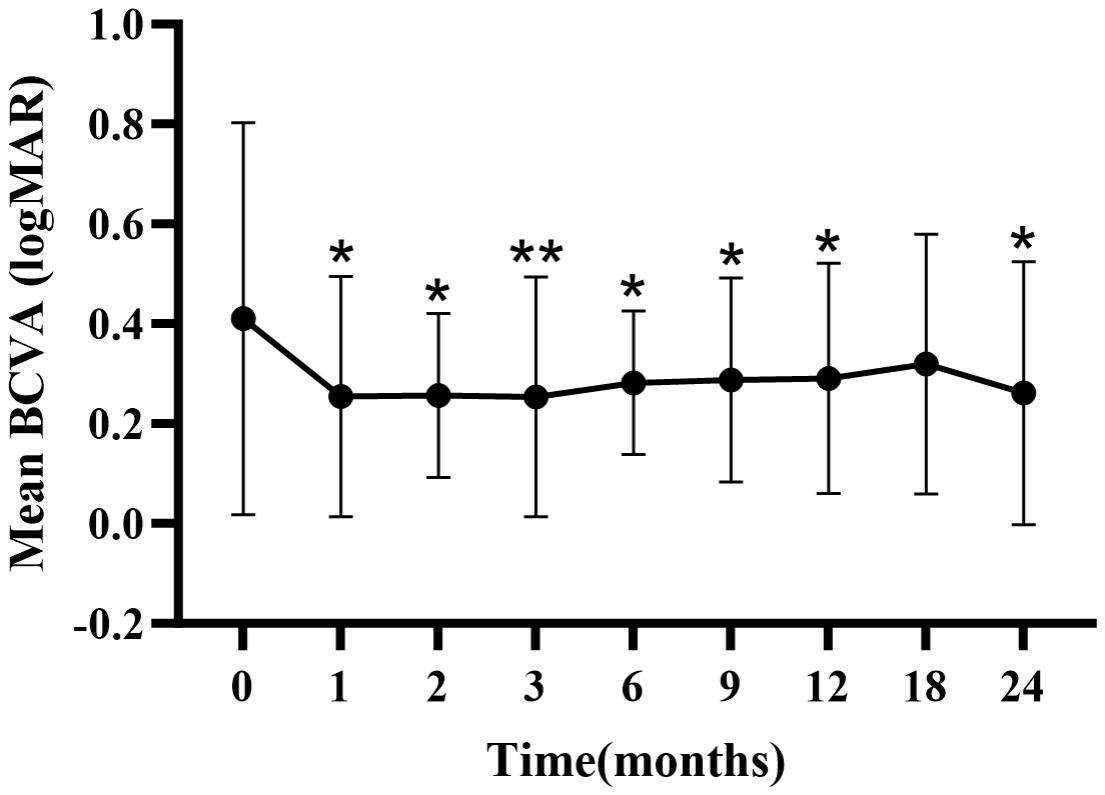
Mean ± standard deviation logMAR BCVA from baseline to the 24-month follow-up. *p < 0.05 vs. baseline; **p < 0.001 vs. baseline. BCVA, best-corrected visual acuity; logMAR, logarithm of the minimum angle of resolution.

There were significant improvements in mean BCVA at 1-, 2-, 3-, 6-, 9-, 12-, and 24-months compared to baseline (*p* < 0.05; Figure 1; Supplementary Table S1), except at the 18-month follow-up.

### Changes in CMT

**Figure 2:**
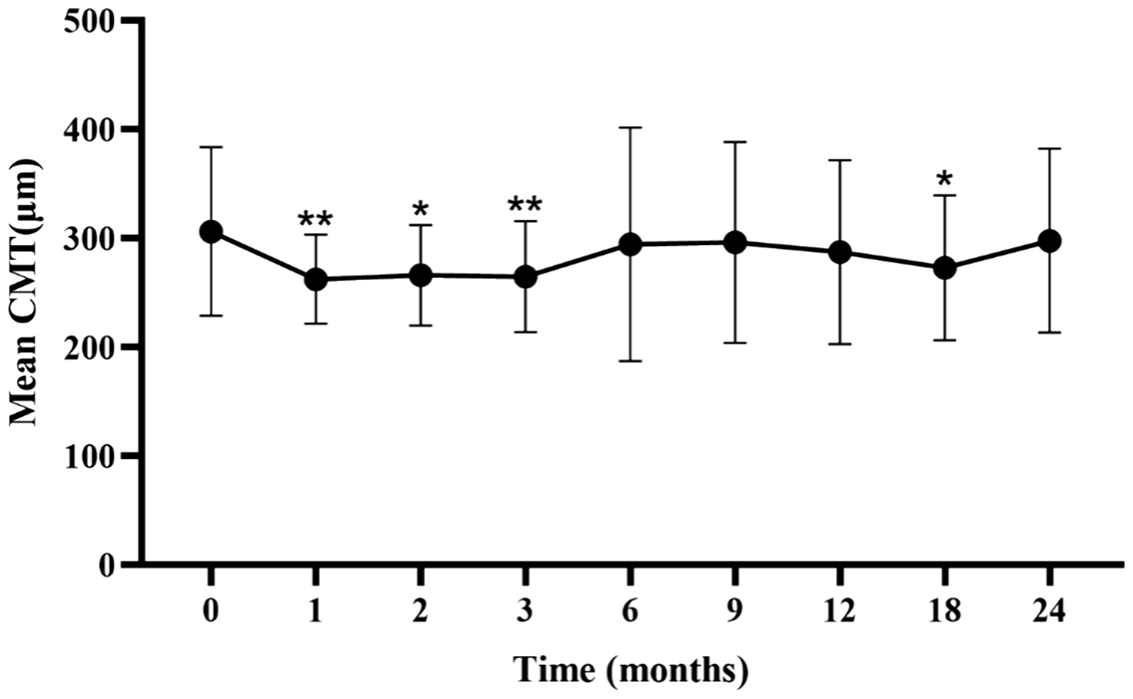
Mean ± standard deviation CMT from baseline to the 24-month follow-up. *p < 0.05 vs. baseline; **p < 0.001 vs. baseline. CMT, central macular thickness.

The mean CMT significantly decreased from baseline during the first 3 months (*p* < 0.05; Figure 2; Supplementary Table S2). A slight increase in CMT was observed from 6 to 24 months, with no significant difference from baseline at 6 months.

### Improvement in DRSS scores

**Figure 3:**
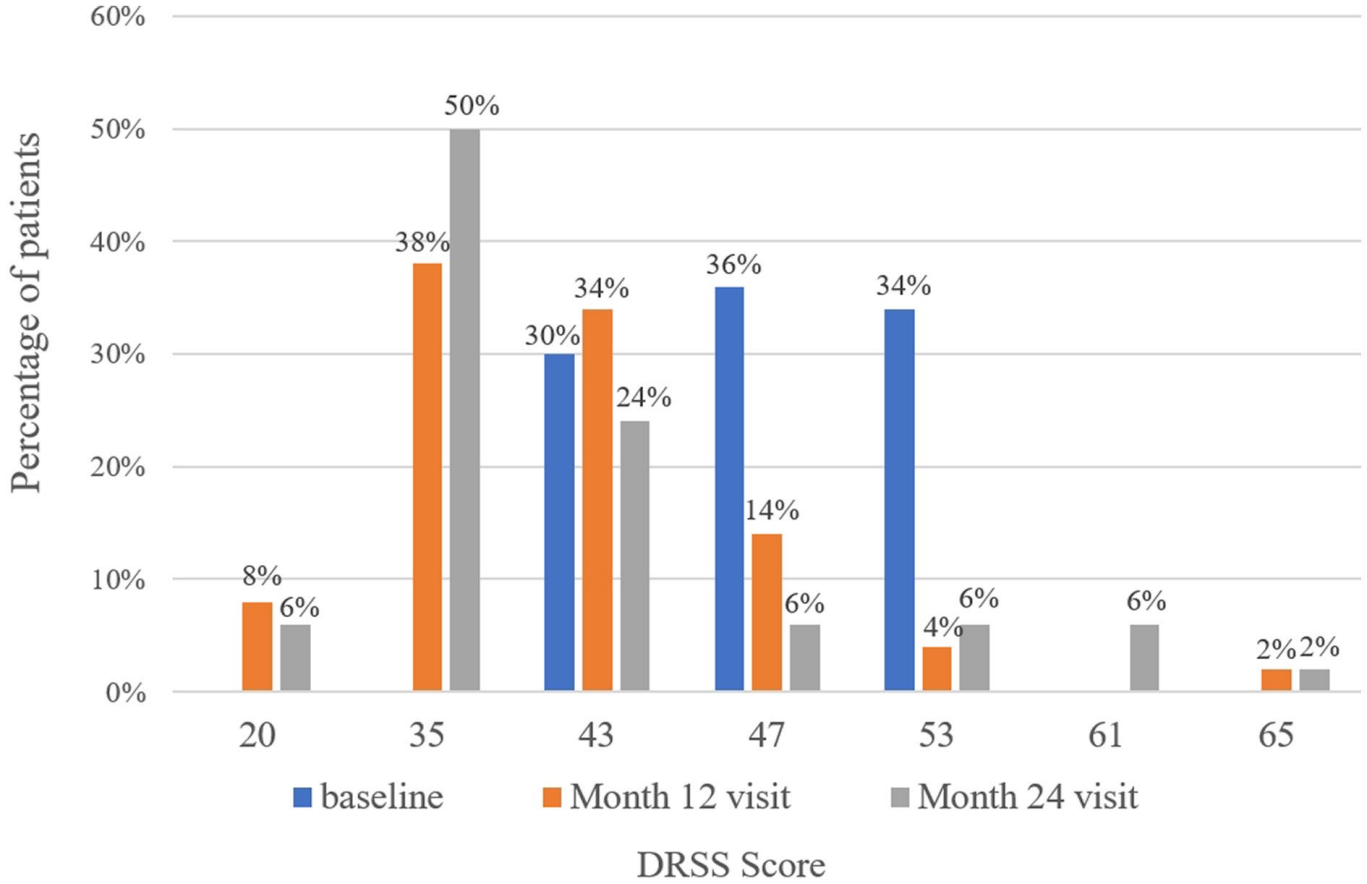
Distribution of patients with different Diabetic Retinopathy Severity Scale scores at baseline, month 12, and month 24.

**Figure 4:**
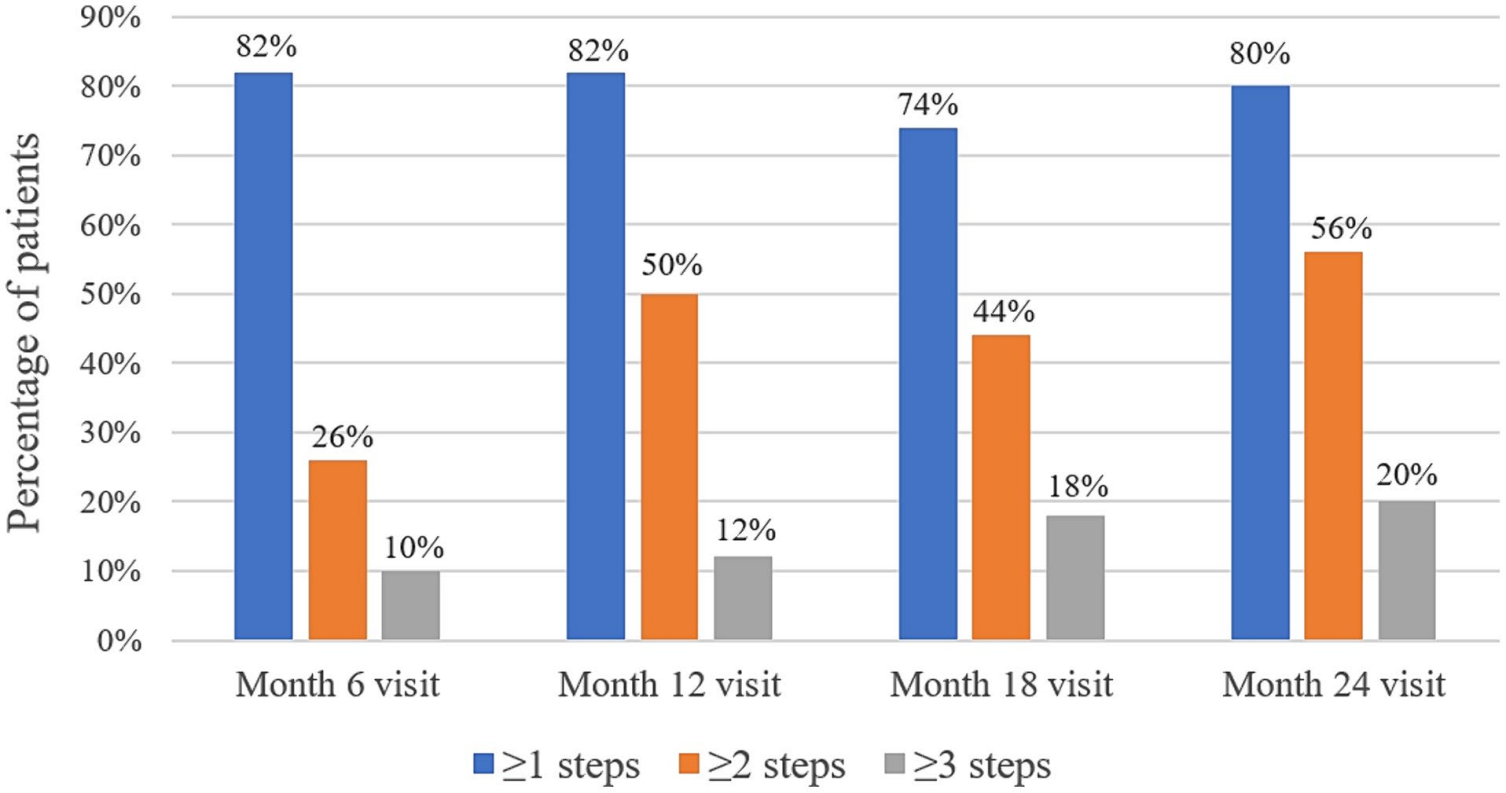
Percentage of patients with an improvement of 1 step or more, 2 steps or more, and 3 steps in their Diabetic Retinopathy Severity Scale scores at month 6, month 12, month 18, and month 24.

**Figure 5:**
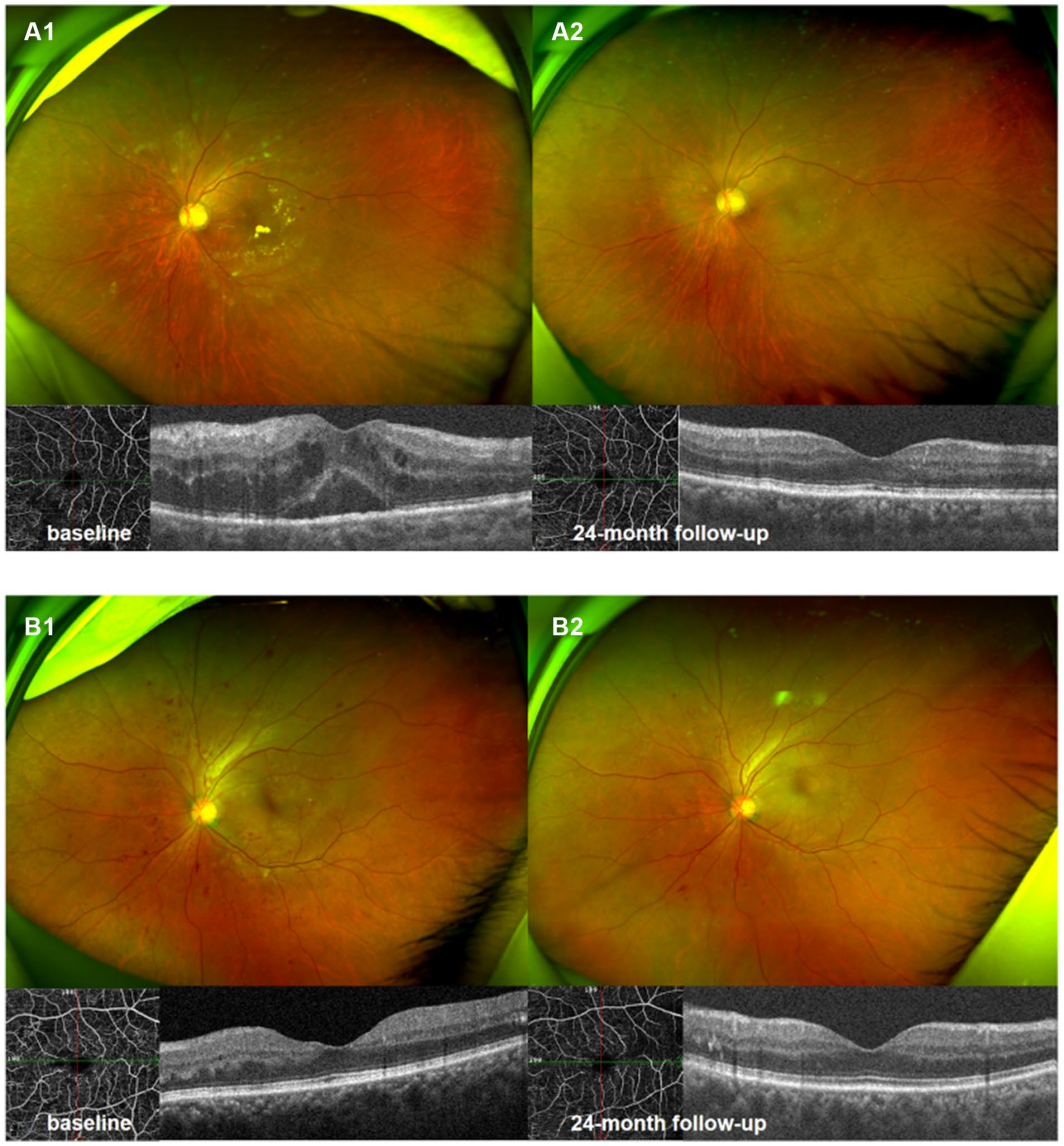
Ultrawide-field fundus photography and optical coherence tomography 6 × 6 mm scan of NPDR eye accompanied with DME **(A1,A2)**, and NPDR eye without DME **(B1,B2)**. Compared with baseline **(A1,B1)**, there were significant improvement in DRSS scores, decrease in hemorrhagic and hard exudates area in 24th month following **(A2,B2)**.

At baseline, the DRSS scores were distributed as follows: 43 in 15 patients (30%), 47 in 18 patients (36%), and 53 in 17 patients (34%) (Figure 3). At the 12-month follow-up, DRSS scores had regressed by at least one step in 41 eyes (82%), by two steps in 25 eyes (50%), and by three steps in 6 eyes (12%). The scores remained stable in 8 eyes (16%), while one eye worsened, developing vitreous hemorrhage. By month 24, DRSS scores had regressed by at least one step in 40 eyes (80%), by two steps in 28 eyes (56%), and by three steps in 10 eyes (20%) (Figure 4), with stability observed in 6 eyes (12%). However, scores worsened in 4 eyes. The DRSS scores at each follow-up interval demonstrated statistically significant improvement from baseline (*p*<0.05; Figure 5; Supplementary Table S3).

### Changes in HE

**Figure 6:**
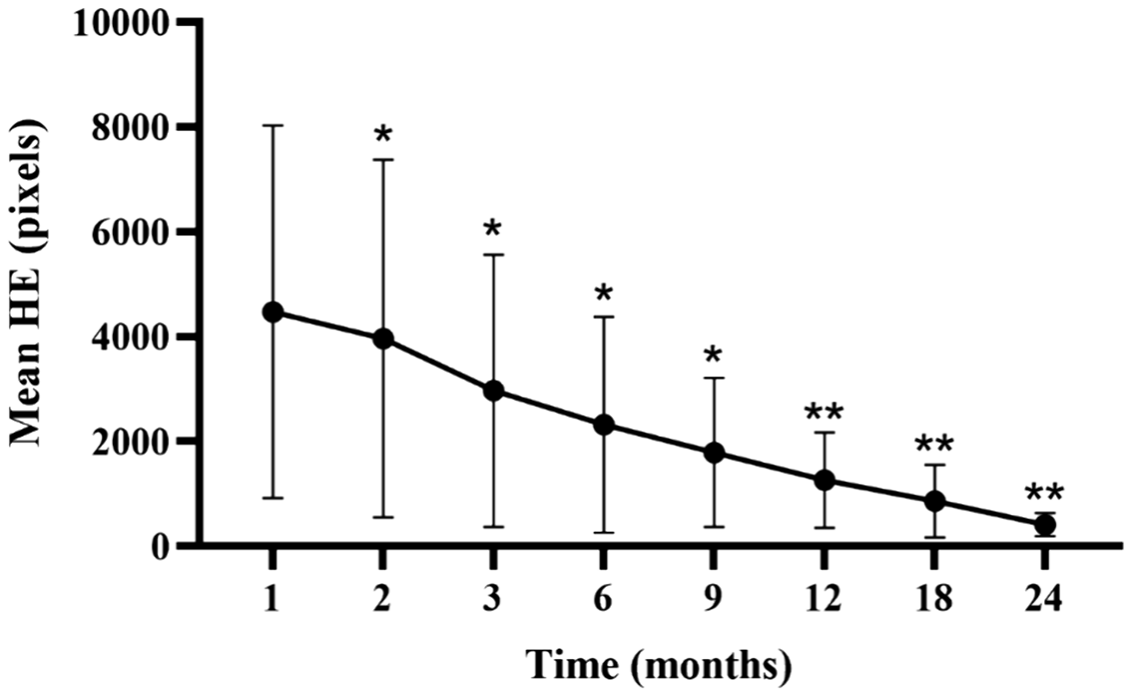
Mean ± standard deviation HE area from 1-month to 24-month follow-up time points. *p < 0.05 vs. baseline; **p < 0.001 vs. baseline. HE, hard exudate.

Analysis of the lesion characteristics in eyes with NPDR identified 15 eyes with HE in the macular vascular arch area prior to treatment, representing 30% of the study cohort. Additionally, 15 of the 27 eyes (55.56%) with DME exhibited varying extents of HE in the same region. Comparisons of the HE areas at each follow-up time point with those at the first month revealed statistically significant reductions (*p*<0.05; Supplementary Table S4; Figure 6). We hypothesized that the persistence of HE within the macular vascular arch was hypothesized with recurrent DME. These findings suggest that early intensive intravitreal injections of conbercept significantly diminished the HE areas.

### Adverse events

Severe ocular adverse events are detailed in Table 2. Twelve eyes (24%) with NPDR progressed to PDR, whereas 16 eyes (69.57%) developed new-onset DME. All eyes with severe DR progression underwent PRP, 6 of which also experienced vitreous hemorrhage and received PRP during the pars plana vitrectomy (PPV) period, followed by an IVC immediately post-surgery. This deterioration might be attributed to the accelerated progression of DR in six patients with chronic renal insufficiency. For the eyes with newly diagnosed DME, the existing monthly IVC regimen was maintained. One eye required local retinal laser photocoagulation due to non-perfusion areas identified during fundus fluorescein angiography. PPV and internal limiting membrane (ILM) peeling were undertaken in one eye with a macular epiretinal membranes, respectively. There were no reports of iatrogenic cataract, endophthalmitis, occlusive retinal vasculitis, ocular inflammation, or persistent ocular hypertension following the injections.

**Table 2:** Severe ocular adverse events and treatment.

Characteristic	*n* (%)	
	Total	Therapy
*PDR progression*		
Vitreous hemorrhage	6 (12)	PPV + PRP + IVC
Proliferative diabetic retinopathy	6 (12)	PRP
Neovascular glaucoma	0	–
New-onset DME in eyes without DME at baseline	16 (69.57)	IVC
Non-perfusion area (FFA)	1 (2)	Local laser photocoagulation
Macular epiretinal membranes	1 (2)	PPV + ILM peeling
Iatrogenic cataract	0	–
Endophthalmitis	0	–
occlusive retinal vasculitis	0	–
ocular inflammation	0	–
Persistent ocular hypertension	0	–

DR, diabetic retinopathy; DME, diabetic macular edema; FFA, fundus fluorescent angiography; PPV, pars plana vitrectomy; PRP, panretinal laser photocoagulation; IVC, intravitreal injection of conbercept; ILM, internal limiting membrane.

## Discussion

When NPDR progresses to PDR, several severe complications can arise from neovascularization, including vitreous hemorrhage, tractional retinal detachment, and neovascular glaucoma. These complications significantly impair visual acuity and detrimentally impact quality of life. Although PPV combined with PRP is an effective treatment for PDR, it may lead to irreversible retinal damage due to fibrosis and contraction, often resulting in permanent vision loss and an increased management burden. Therefore, exploring treatment options for DR at early stages and strategies to prevent or delay the transition from NPDR to PDR is critical.

Compared to VEGF-trap agents such as aflibercept, conbercept is a novel anti-VEGF drug that includes the fourth binding domain of VEGFR-2, enhancing the stability of the receptor–ligand complex and extending the drug’s half-life (19). As a result, conbercept demonstrates high affinity and prolonged efficacy. Our findings suggest that IVC treatment for patients with NPDR effectively improves BCVA and DRSS levels at the 24-month follow-up, as well as reduces CMT and HE areas.

Although NPDR is characterized by significant pathological changes, patients without DME often do not report symptoms. The risk of progressing from severe NPDR to PDR within 1 year was 15%, increasing to 60% over 3 years. Patients with very severe NPDR face a 45% likelihood of advancing to PDR within 1 year (20). In the PROTOCOL W study, the cumulative probability of developing PDR within 2 years was 13.5% in the aflibercept group compared to 33.2% in the sham group (14). While our study found that 24% of patients with moderate to severe NPDR treated with IVC developed PDR within 2 years—a rate slightly higher than previous studies—this increase is attributed to the accelerated progression of DR in six patients with chronic renal insufficiency. Excluding these patients, the proportion of patients progressing to PDR during the observation period was 10%. To prevent irreversible visual impairment, close monitoring of patients with NPDR is essential. A *post hoc* analysis of 748 DME eyes from the VIVID and VISTA studies indicated that patients receiving intravitreal aflibercept had superior BCVA outcomes compared to those undergoing laser photocoagulation. Specifically, improvements in the DRSS of ≥2 steps were observed in 13% of eyes with a baseline DRSS score ≤ 43, 25.8% with a score of 47, and 64.5% with a score ≥ 53 (15). In the PANORAMA study, NPDR patients without DME and with DRSS scores of 47–53 were treated with aflibercept administered every 16 weeks. This regimen sustained improvements in DRSS levels, with 65.2% of patients showing an improvement of ≥2 steps at week 52, which persisted at 62.2% through week 100 (13). In this study, 56% of NPDR patients achieved a DRSS regression of ≥2 at the 24th month following IVC therapy, demonstrating significant reductions in the severity of diabetic retinopathy, which aligns well with prior findings. PROTOCOL W compared aflibercept administered every 16 weeks against a sham treatment to prevent PDR and DME in a similar NPDR cohort (DRSS level of 43–53 and BCVA ≥0.8) (14). It was found that a significantly higher proportion of patients improved by ≥2 DRSS steps with aflibercept compared to the sham. Additionally, the incidence of PDR or central-involved DME with vision loss was significantly lower in the aflibercept group, maintaining this advantage for up to 2 years post-treatment.

HE appear as yellow and white deposits on the retina, primarily composed of lipids and proteins such as fibrinogen and albumin, predominantly located in the outer plexus layer. Histopathological evaluations have revealed that diffuse lipid accumulation, cholesterol esterification, apolipoprotein B, and macrophages are typically found surrounding the compromised blood vessels within retinal HE lesions (21). Although HE sometimes resolves spontaneously, it can progress to fibrotic lesions, which may lead to severe vision loss, particularly if occurring in the macular fovea (22). In the Phase III RISE and RIDE trials, monthly injections of ranibizumab significantly mitigated intraretinal HE in DME eyes compared to sham injections, although the reduction in HE area was more gradual compared to the rapid response observed in treating DME (23). Another similar study focused on the efficacy and safety of conbercept for severe NPDR and found significant improvements in HE area during 9 ~ 12 months of follow-up (24). Our findings demonstrate a significant reduction in HE area at all follow-up intervals in patients with NPDR; however, additional long-term studies with larger cohorts are required to further explore the pathological mechanisms and efficacy of IVC therapy in treating HE.

Our study has several limitations. Firstly, the validity of our results would benefit from a broader case base, particularly focusing on the effects of well-managed systemic diseases on the outcomes. Secondly, the observation period was insufficient to assess long-term outcomes and sustained compliance with anti-vascular endothelial growth factor (anti-VEGF) therapy in NPDR eyes, and the cost-effectiveness ratio presents another challenge to be addressed. Thirdly, it remains unclear whether improvements in the DRSS induced by anti-VEGF therapy are associated with retinal reperfusion.

## Conclusion

In conclusion, our study has shown that early intensive intravitreal injections of conbercept significantly reduce DRSS scores and improve BCVA, as well as decrease CMT and HE areas in patients with moderate to severe NPDR. These results suggest that conbercept effectively slows the progression of NPDR to PDR. Nevertheless, extended follow-up studies spanning at least 5 years are essential to fully ascertain the long-term visual benefits of early anti-VEGF therapy in this patient population.

## Data availability statement

The original contributions presented in the study are included in the article/Supplementary material, further inquiries can be directed to the corresponding authors.

## Ethics statement

The studies involving humans were approved by the Ethics Review Committee of the First Affiliated Hospital of Kunming Medical University. The studies were conducted in accordance with the local legislation and institutional requirements. Written informed consent for participation was not required from the participants or the participants’ legal guardians/next of kin in accordance with the national legislation and institutional requirements.

## Author contributions

LS: Writing – review & editing, Writing – original draft, Investigation, Data curation. YZ: Writing – original draft, Data curation. ZG: Writing – review & editing, Methodology. QL: Writing – original draft, Data curation. MD: Writing – original draft, Investigation. WY: Writing – original draft, Investigation, Data curation. QZ: Writing – original draft, Investigation. DL: Writing – review & editing, Supervision, Project administration. YH: Writing – review & editing, Visualization, Supervision. LY: Writing – review & editing, Validation, Supervision, Resources, Project administration, Funding acquisition.
