# Supplementary material for: Efficacy and safety of intravitreal injection of conbercept for moderate to severe nonproliferative diabetic retinopathy
Source: Front Med (Lausanne). 2024 May 23;11:1394358. doi: 10.3389/fmed.2024.1394358 (PMC11153662; doi:10.3389/fmed.2024.1394358)
Supplement: Supplementary file 1 [file Table_1.DOC]

**Supplementary Information:**

**Efficacy and Safety of Intravitreal Injection of Conbercept for Moderate to Severe** **Nonproliferative Diabetic Retinopathy**

Lu Shen^1#^, Yuxiang Zheng^1#^, Zelan Gao^2^, Qirui Li^1^, Min Dai^1^, Wenchang Yang^1^, Qiying Zhang^1^, Dongli Li^1^, Yijun Hu^3*^, Ling Yuan^1*^

^1^ Department of Ophthalmology, The First Affiliated Hospital of Kunming Medical University, No. 295 Xichang Road, Kunming, 650032, China;

^2^ Department of Ophthalmology, Yan’an Hospital of Kunming City, No. 245 East Renmin Road, Kunming, 650051, China;

^3^ Guangdong Eye Institute, Department of Ophthalmology, Guangdong Provincial People’s Hospital, Guangdong Academy of Medical Sciences, Guangzhou, 510080, China

*** Correspondence to:** Prof. Ling Yuan, Department of Ophthalmology, The First Affiliated Hospital of Kunming Medical University, 295 Xichang Road, Kunming, China; Fax: 650032; Email: yuanling8061@163.com. Dr. Yijun Hu, Guangdong Eye Institute, Department of Ophthalmology, Guangdong Provincial People’s Hospital; Guangdong Academy of Medical Sciences, No. 106 Zhongshan Er Road, Yuexiu District, Guangzhou 510080, China. Email: [huyijun2014@163.com](mailto:huyijun2014@163.com).

# These authors contributed equally to the drafting of the manuscript and reviewed the literature and therefore can be considered as first co-authors.

**Table S1**. Best-corrected visual acuity (logMAR) values over time in patients with nonproliferative diabetic retinopathy who received intravitreal injections of conbercept.

|  | Before treatment | | 1 month | 2 months | 3 months | 6 months | 9 months | 12 months | | 18 months | 24 months | |
| --- | --- | --- | --- | --- | --- | --- | --- | --- | --- | --- | --- | --- |
| n=50 | 0.41±0.39 | | 0.25±0.26 | 0.24±0.16 | 0.24±0.24 | 0.28±0.15 | 0.27±0.20 | | 0.26±0.22 | 0.30±0.25 | | 0.23±0.20 |
| t | - | | 2.931 | 3.200 | 3.611 | 2.201 | 2.598 | | 2.394 | 1.784 | | 3.137 |
| P | - | 0.006 | | 0.003 | 0.001 | 0.035 | 0.014 | | 0.022 | 0.083 | | 0.004 |

Values are shown as mean ± standard deviation.

P value vs. before treatment.

Abbreviations: logMAR, logarithm of the minimum angle of resolution.

**Table S2.** Central macular thickness (μm) values over time in patients with nonproliferative diabetic retinopathy who received intravitreal injections of conbercept.

|  | Before treatment | | 1 month | 2 months | 3 months | 6 months | 9 months | 12 months | | 18 months | 24 months | |
| --- | --- | --- | --- | --- | --- | --- | --- | --- | --- | --- | --- | --- |
| n=50 | 306.22±77.40 | | 261.24±42.19 | 262.69±46.44 | 262.54±50.48 | 296.14±112.20 | 297.75±95.98 | | 288.39±87.54 | 270.60±68.07 | | 297.97±88.15 |
| t | - | | 4.655 | 3.373 | 3.668 | 0.477 | 0.423 | | 1.076 | 2.870 | | 0.881 |
| P | - | <0.001 | | 0.002 | <0.001 | 0.636 | 0.675 | | 0.289 | 0.007 | | 0.385 |

Values are shown as mean ± standard deviation.

P value vs. before treatment.

**Table S3.** Diabetic Retinopathy Severity Scale scores over time in patients with nonproliferative diabetic retinopathy who received intravitreal injections of conbercept.

|  | Before treatment | | 1 month | 2 months | 3 months | 6 months | 9 months | 12 months | 18 months | 24 months |
| --- | --- | --- | --- | --- | --- | --- | --- | --- | --- | --- |
| Z | | - | −3.223 | −4.003 | −4.224 | −5.051 | −4.878 | −4.522 | −3.579 | −3.646 |
| P | | - | 0.001 | <0.001 | <0.001 | <0.001 | <0.001 | <0.001 | <0.001 | <0.001 |

P value vs. before treatment.

**Table S4.** Hard exudate areas over time among patients with nonproliferative diabetic retinopathy who received intravitreal injections of conbercept.

|  | Area of HE (pixels) | Mean range | t | P |
| --- | --- | --- | --- | --- |
| 1 month | 3554.50±1927.24 | - | - | - |
| 2 months | 2906.70±1590.50 | 647.80±769.76 | 2.661 | 0.026 |
| 3 months | 2163.00±1445.04 | 1391.50±1525.90 | 2.884 | 0.018 |
| 6 months | 1779.50±1401.78 | 1775.00±1361.79 | 4.122 | 0.003 |
| 9 months | 1369.78±832.61 | 2413.00±1475.46 | 4.906 | 0.001 |
| 12 months | 1005.33±570.58 | 2777.44±1496.90 | 5.566 | <0.001 |
| 18 months | 664.22±229.88 | 3118.56±1798.83 | 5.201 | <0.001 |
| 24 months | 385.11±148.61 | 3397.67±1880.74 | 5.420 | <0.001 |

Values are shown as mean ± standard deviation

P value vs. 1st month.
